# Low density lipoprotein cholesterol control status among Canadians at risk for cardiovascular disease: findings from the Canadian Primary Care Sentinel Surveillance Network Database

**DOI:** 10.1186/s12944-015-0056-8

**Published:** 2015-06-24

**Authors:** Erfan Aref-Eshghi, Jason Leung, Marshall Godwin, Pauline Duke, Tyler Williamson, Masoud Mahdavian, Shabnam Asghari

**Affiliations:** Memorial University of Newfoundland, St. John’s, Newfoundland and Labrador Canada; University of Calgary, Calgary, Alberta Canada; University of Ottawa, Ottawa, Ontario Canada; Department of Family Medicine, Faculty of Medicine, Memorial University of Newfoundland, Primary Healthcare Research Unit, Agnes Cowan Hostel, Room 425, Health Sciences Centre, 300 Prince Philip Drive, St. John’s, NL A1B 3V6 Canada

**Keywords:** Dyslipidemia, Canada, LDL-C control, CPCSSN

## Abstract

**Background:**

To determine the prevalence of uncontrolled LDL-C in patients with high cardiovascular disease (CVD) risks across Canada and to examine its related factors.

**Methods:**

Non-pregnant adults >20 years-old, who had a lipid test completed between January 1, 2009 and December 31, 2011 and were included in the Canadian Primary Care Surveillance Network (CPCSSN) database were studied. The Framingham-Risk-Score was calculated to determine the risk levels. A serum LDL-C level of >2.0 mmol/L was considered as being poorly controlled. Patients with a previous record of a cerebrovascular accident, peripheral artery disease, or an ischemic heart disease were regarded as those under secondary prevention. Logistic regression modeling was performed to examine the factors associated with the LDL-C control.

**Results:**

A total of 6,405 high-risk patients were included in the study and, of this population, 68 % had a suboptimal LDL-C, which was significantly associated with the female gender (OR: 3.26; 95 % CI: 2.63–4.05, *p* < 0.0001) and no medication therapy (OR: 6.31, 95 % CI: 5.21–7.65, *p* < 0.0001). Those with comorbidities of diabetes, hypertension, obesity, and smokers had a better LDL-C control. Rural residents (OR: 0.64, 95 % CI: 0.52–0.78, *p* < 0.0001), and those under secondary prevention (OR: 0.42; 95 % CI: 0.35–0.51, *p* < 0.0001), were also more likely to have a better LDL-C control.

**Conclusion:**

A high proportion of high-cardiac risk patients in Canadian primary care settings have suboptimal LDL-C control. A lack of medication therapy appears to be the major contributing factor to this situation.

## Introduction

Cardiovascular diseases (CVD) are the leading cause of death worldwide with more than 17.3 million deaths in 2008 according to a WHO report [1]. In Canada, CVD is the main cause of death at 32 % and, after musculoskeletal diseases, has the highest economic burden of disease [2]. Hyperlipidemia, defined as abnormal blood lipid levels including elevated total cholesterol, low-density lipoprotein cholesterol (LDL-C), triglycerides and decreased high-density lipoprotein cholesterol, is well established as a major risk factor to CVD [3]. Similar to many other countries, the management of dyslipidemia, in order to reduce the CVD risk in a clinical setting in Canada, is conducted following the instructions by the available guidelines, including those by the Canadian Cardiovascular Society [4]. These guidelines are modeled after the Framingham Risk Assessment and target specific lipid goals based on the cardiovascular risk. Overall, patients with a higher cardiovascular risk are treated more aggressively than those with a lower risk. Thus, patients with a 10-year cardiovascular risk of >20 % will have an LDL-C goal of ≤2 mmol/L, while patients with a 10-year cardiovascular risk of <5 % may or may not be treated depending on their initial LDL-C levels. The LDL-C reduction is primarily administered by medication therapy, although diet and physical activity have also been suggested to be effective [5]. Statins are the most commonly used medications for lowering LDL-C levels. Every 1.0 mmol/L reduction in LDL-C by statins has been reported to be associated with a ~23 % relative risk reduction in major vascular events over five years of treatment and follow-up [6]. A 2.0 mmol/L absolute reduction, or a 50 % relative reduction in LDL-C, provides optimal benefits in CVD risk management; thus, those with a higher CVD risk are more likely to benefit from the management of dyslipidemia [4]. Lipid level targets, however, are sometimes challenging to achieve. While various reports suggest that LDL-C levels are not lowered to target a significant portion of the high-risk populations [6–8], there is no recent report available on the status of LDL-C control among Canadian patients at risk. Canadian guidelines for the diagnosis and treatment of dyslipidemia [4] recommend an LDL-C target goal of ≤2.0 mmol/L for individuals with a high 10-year risk of a cardiovascular event. In the present study, we determine the prevalence of suboptimal LDL-C (LDL-C > 2 mmol/L) in high-risk individuals in a primary care setting in Canada. As well, we examine the factors associated with the rates of LDL-C control.

## Results

**Fig. 1:**
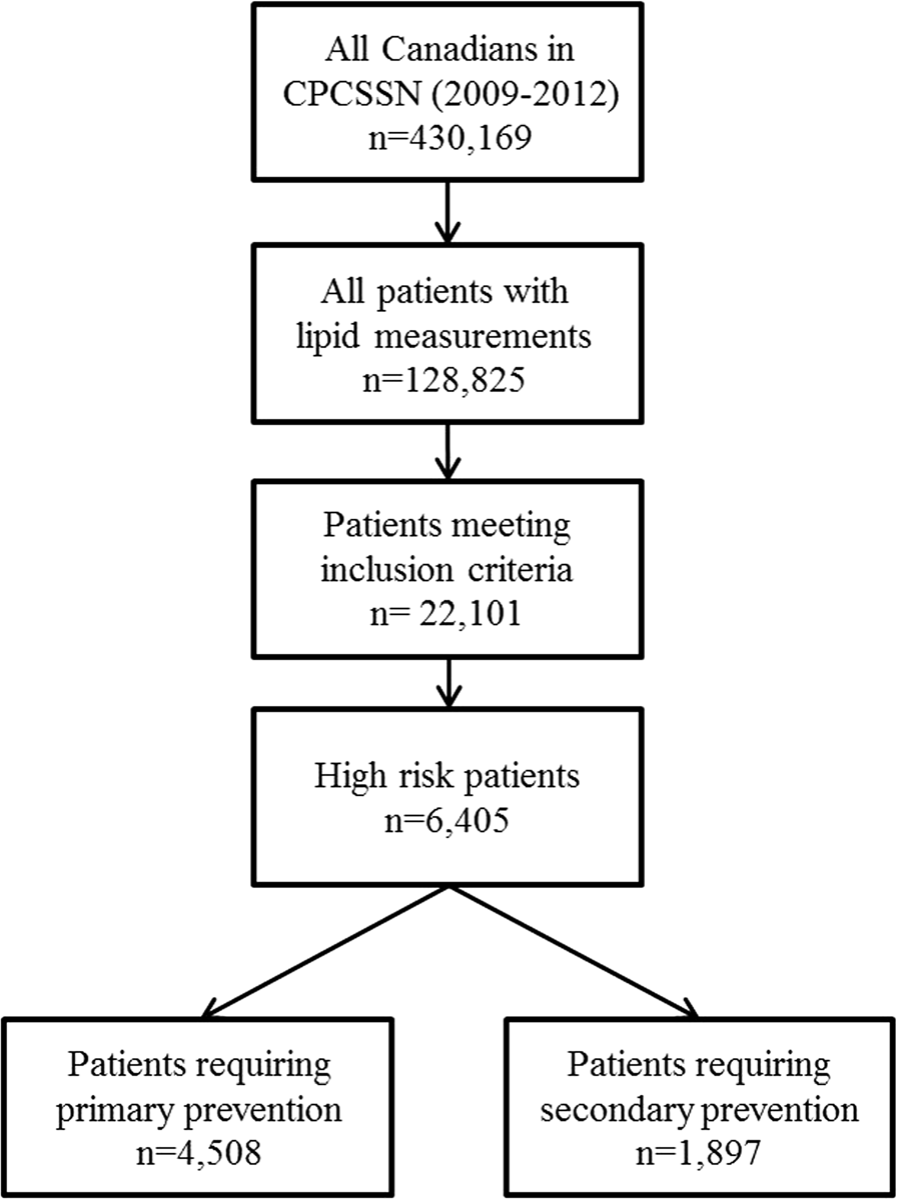
The flowchart of the study

Among the 22,101 individuals who met the inclusion criteria (Fig. 1), 6,405 (29 %) were classified as high risk, and were included in the analysis (aged 71.9 ± 10.3, 23 % female, 24 % rural residence). Almost half of the population (46 %) was classified as non-medication users, whereas 12 % and 41.5 % were previous and current users, respectively. The prevalence of those with uncontrolled LDL-C was 68 % and 29 % were determined to be in need of secondary prevention (Fig. 1).

As seen in Table 1, the prevalence of uncontrolled LDL-C gradually decreases with age. High-risk females have a higher rate of uncontrolled LDL-C (77 %) compared to high-risk males with 66 %. Whereas 89 % of non-medication users have sub-optimal LDL-C, the rates are much lower to about 50 % only among previous and current medication users. The rates are approximately 30 % higher among those under primary prevention than those individuals under secondary prevention. Diabetic high-risk patients, however, had a 30 % lower rate of uncontrolled LDL compared to non-diabetics (Table 1).

**Table 1:** Prevalence of uncontrolled LDL among high-risk patients in Canadian EMR primary care settings by category of risk factors

Risk factors	High-risk patients	
	Total (*n* = 6405)	Uncontrolled LDL (68.6 %)
Gender		
Male	4931	66.0 %
Female	1474	77.3 %
Age		
< 40	2	100.0 %
40–50	72	85.7 %
50–60	599	81.2 %
60–70	1726	72.5 %
70–80	2185	66.3 %
> 80	1821	63.0 %
Smoking status		
Current smokers	1396	73.5 %
Past smokers	2850	64.1 %
Non-smokers	2159	71.4 %
Diabetes		
Diabetics	2715	51.6 %
Non-diabetics	3690	81.0 %
Hypertension		
Hypertensive	3911	66.9 %
Non-hypertensive	2494	71.2 %
Obesity		
Normal/underweight	1218	70.5 %
Overweight	2754	70.3 %
Obese	2433	61.1 %
Lipid-lowering medication use		
Non-users	2958	89.5 %
Previous users	789	50.4 %
Current users	2658	50.6 %
Place of residence		
Rural	4754	70.5 %
Urban	1501	67.8 %
Type of prevention		
Primary	4508	77.3 %
Secondary	1897	47.9 %

Table 2 represents the charachteristics of the study population among those with uncontrolled and controlled levels of LDL-C. Table 3 shows the results of the logistic regression model in the high-risk population, adjusting for gender, age, place of residence, comorbidities, and type of prevention. As observed, women are more likely to have an LDL-C above 2.0 mmol/L than men (OR: 3.26; 95 % CI: 2.63–4.05, *p* < 0.0001), and aging is significantly associated with the lower odds of having an uncontrolled LDL-C (OR: 0.95, 95 % CI: 0.94–0.96, *p* < 0.0001). Comorbidities were associated with a lower rate of suboptimal LDL-C (obesity [OR: 0.62, 95 % CI: 0.46–0.84, *p* = 0.002], current smokers [OR: 0.44, 95 % CI: 0.33–0.59, *p* < 0.0001], diabetes [OR: 0.13, 95 % CI: 0.11–0.16, *p* < 0.0001], and hypertension [OR: 0.72, 95 % CI: 0.61–0.86, *p* < 0.0001]). Individuals who were not receiving any lipid-lowering medication had a 6.31 (95 % CI: 5.21–7.65) fold increased odds of having an LDL-C above 2 mmol/L compared to current medication users (*p* < 0.0001). Rural residents had a better LDL-C control (OR: 0.64, 95 % CI: 0.52–0.78, *p* < 0.0001) compared to urban dwellers. Patients who were being treated for the purpose of secondary prevention were better controlled in terms of LDL-C (OR: 0.42; 95 % CI: 0.35–0.51, *p* < 0.0001) than patients who were being treated for the purpose of primary prevention.

**Table 2:** CVD associated factors among high-risk patients with and without controlled levels of LDL-C in Canadian EMR primary care settings

Risk factors	Controlled LDL	Uncontrolled LDL	*P*-value
Age (Mean ± SD)	73.80 ± 9.57	71.27 ± 10.4	<0.0001
Gender (female)	17.5 %	26.5 %	<0.0001
Body mass index (Mean ± SD)	29.93 ± 5.84	28.85 ± 5.39	<0.0001
Diabetes	65 %	32 %	<0.0001
Hypertension	64 %	59 %	<0.0001
Smoking			
Non-smokers	30.8 %	35.2 %	<0.0001
Past smokers	50.9 %	41.6 %	
Current smokers	18.1 %	23.1 %	
Lipid-lowering medication use			
Non-user	15.5 %	60.4 %	<0.0001
Previous user	19.3 %	8.9 %	
Current user	65.1 %	30.6 %	
Residence (rural)	22.3 %	25.1 %	0.055
Prevention (secondary)	49.00 %	20.60 %	<0.0001

**Table 3:** Multivariate logistic regression model for uncontrolled LDL among high risk patients in Canadian EMR primary care settings^a^

Risk factors (reference group)	Odds ratio (95%CI)	*P*-value
Gender (males)	3.26 (2.63–4.05)	<0.0001
Age	0.95 (0.94–0.96)	<0.0001
Obesity (BMI ≤25)		
Overweight	1.05 (0.77–1.42)	0.775
Obese	0.62 (0.46–0.84)	0.002
Smoking status (nonsmokers)		
Past smokers	0.85 (0.71–1.01)	0.062
Current smokers	0.44 (0.33–0.59)	<0.0001
Diabetes	0.13 (0.11–0.16)	<0.0001
Hypertension	0.72 (0.61–0.86)	<0.0001
Lipid-lowering medication (current users)		
Previous users	1.28 (1.02–1.61)	0.035
Non-users	6.31 (5.21–7.65)	<0.0001
Place of residence (urban)	0.64 (0.52–0.78)	<0.0001
Type of prevention (primary)	0.42 (0.35–0.51)	<0.0001

^a^ Patients with diabetes and hypertension were compared to those without diabetes or hypertension, respectively

## Discussion

Our study is the first of its kind to assess the status of LDL-C control in a targeted population for CVD primary and secondary prevention in a primary care setting across Canada. We used a cross-sectional study design using the electronic medical records of the Canadian population under primary care that is at a high risk for CVD. The information for risk calculation was only available for less than a quarter of the subjects who had a record of completing a lipid test; this is mainly due to the missing information on smoking (~70 %), suggesting that FRS is not measured for a large proportion of patients in primary care despite the recommendations by the guidelines. It also indicates that the smoking status, a well-known risk factor of CVD, is not regularly tracked and recorded by family physicians. This raises the question as to what extent the FRS is being practiced and whether it is influencing the primary care practice at all, since many practitioners might use other criteria as an indication of LDL-C lowering treatment. Nevertheless, according to our findings, approximately three-quarters of the high-risk population have poorly controlled LDL-C. The status of control is better among men, the elderly, rural residents, those under secondary prevention management, those taking lipid modifying agents, and those with comorbidities. In our study, lipid modifying agents have the greatest effect on the status of LDL-C control. Non-medication users have 6.3 fold increased odds of having poorly controlled LDL-C compared to current users. Previous medication users were not significantly different compared with non-users, which suggests that the effect of lipid lowering medications does not endure three months from cessation. Approximately 90 % of the lipid lowering medication users were on statins; similar results were identified where we limited the study to statin users. Therefore, it could be hypothesized that a lack of medication therapy may be a major contributor to a low rate of LDL-C control in Canada, which may possibly be related to a lack of updated knowledge among physicians or the unwillingness of patients to participate in medication therapy. Since half of the current medication users still have suboptimal levels of LDL-C, other factors, such as a lack of physical activity and appropriate dieting among the patients, as well as the satisfaction of physicians with LDL-C levels reaching close to the cut-off point, may also be considered. Moreover, of special importance are the status of nutrition and the use of nutraceutical in treating dyslipidemia, given that several studies report that these agents, in addition to statin therapy, need to be considered in treating dyslipidemia [7]. Since we did not have access to this information, our multivariate model cannot predict the effect of these factors.

Although the number of similar studies performed on LDL-C control worldwide is limited and, to our knowledge, no such report is available from Canada, the findings of this study are comparable to those few studies conducted elsewhere. In a Chinese study, only 37 % of patients with Coronary Heart Disease had a desirable LDL-C level [8]. This figure was lower among very high-risk patients and differed among the two genders and various age groups. In a Brazilian study, only 30 % of the patients obtained the advisable LDL-C levels and the rates were significantly lower among those with poor income [9]. A recent study of 17.5 million statin treated high-risk adults in the United States [10] found that ~30 % of the statin-treated patients had not reached the recommended LDL-C levels of 100 mg/dl (2.5 mmol/L), and ~75 % had an LDL-C >70 mg/dl (1.8 mmol/L) deemed optional according to the American guidelines [11] at the time of the study. These figures would possibly increase if the study had taken into consideration the untreated populations.

The main recommendation by the Canadian guidelines for high-risk patients is to reduce the LDL-C to ≤2.0 mmol/L. Alternatively, an ≥50 % reduction from the baseline LDL-C, in addition to an apoB target level of less than 0.8 g/L, can be implemented. It might be possible that some of the health-care providers follow the alternative recommendations based on which the LDL-C levels do not have to be lowered to 2.0 mmol/L. Otherwise, they might have not found the presented evidence by the guidelines compelling and made an informed valued decision not to initiate pharmacologic treatment. These inconsistencies may account for a portion of those with uncontrolled LDL-C, a subject matter that is not possible to assess merely from electronic medical records, since we did not have access to the baseline levels of lipids at the time the therapy was initiated for the patients.

This study is limited by several factors; i.e., the high risk population was defined according the Canadian Cardiovascular Guidelines at the time of the study (2009) [4]. Recent cardiovascular guidelines still consider FRS ≥20 % as a high risk of CVD; however, other disease conditions, e.g., patients with chronic kidney disease have been added to the high-risk groups and recommended for cardiovascular risk management. The patients in our study are not treated and evaluated according to the new guidelines and thus their status of the newly added high-risk medical conditions is not known.

Among the 128,825 individuals who had lipid testing during the study period, 22,101 (<20 %) individuals had a measure of cardiovascular risk factors within six months of the lipid tests. In order to avoid the possibility of bias due to the unavailable information, we compared the LDL-C levels of those with and without risk classification data. We observed a minimal difference between the two groups suggesting a low possibility of selection bias (uncontrolled LDL-C: 80 vs. 83 %, LDL-C levels: 2.91 vs. 2.87 mmol/dl, respectively).

The cross-sectional nature of the study did not accurately permit the interpretation of the causal and temporal relationships across the variables. As such, the relationship between LDL-C control and its associated factors, including CVD, diabetes and hypertension, should be interpreted with caution in terms of the therapeutic regime suggested by the CVD prevention guidelines for patients with those conditions [4]. Since a similar pattern is observed for aging, obesity and smoking, the latter explanation seems more likely as all of the patients in our study were high risk and most likely under more stringent therapeutic measures. These factors are indicators of higher Framingham-Risk-Scores and it is more likely to draw the attention of the practitioners to such treatments. As well, it was not possible to represent the variability of lipid and risk levels over time, given that they change constantly. We did not have information on medication dosage to determine if the medication users were on adequate dosage; however, the study findings show that lipid-lowering medication users were more likely to attain the lipid goals compared to non-users. There was no information available from the family histories of the patients on the comorbidities, such as Type II Diabetes, which is known to increase the risk of CVDs in the non-diabetes population [12, 13]. Finally, the prevention class categorization may not be ideal, given that the database included information from the year 2006 onward. In that regard, the patients would still be classified into primary prevention, despite the possibility of a disease condition CVED or IHD occurrence before 2006, even though previous studies show that physicians keep updated EMR profiles of those patients at risk of cardiovascular diseases [14].

## Conclusion

A large portion of high-risk patients in Canada have suboptimal LDL-C control. A lack of medication therapy seems to be the most potential contributing factor to this condition, which could result from alternative recommendations or informed decisions by the health-care providers. Our data suggests that there is significant opportunity for improvement. Further studies may also be needed to elucidate other potential causes of uncontrolled LDL-C in Canada, specifically variations in medical practice and patients’ adherence and tolerance. In addition, providers in the health-care system need to identify the barriers to medication adherence and implement new methods to improve medication adherence in high-risk patients.

## Methods

### Study design

This is a cross-sectional study using a secondary analysis of the data from the Canadian Primary Care Sentinel Surveillance Network (CPCSSN) database.

### Data source

CPCSSN is Canada’s first library of digital information based on point-of-care data from primary-care practices. Data from these EMRs is extracted quarterly and uploaded in a de-identified format to both regional and central (pan-Canadian) databases. The database contains a comprehensive record of all diagnoses, health conditions, risk factors, laboratory tests, medication use, as well as other services provided by family physicians in Canada. It is increasingly used for chronic disease surveillance in primary care and also as a tool for conducting primary-care research [15]. At the time of this study, the CPCSSN database included data from close to 600 primary-care clinicians in rural and urban settings across ten Canadian provinces with data on 844,592 individuals [16].

### Study population

The study population included adults at high risk of CVD according to Canadian guidelines at the time of the study [4]. According to these guidelines, the patients with a 10-year risk ≥20 % of developing CVD were considered as high risks and were included in the study.

To identify the high-risk patients, all non-pregnant adults aged 20 years and older who had a lipid profile in their record between January 1, 2009 and December 31, 2011 were identified from the CPCSSN database. The 10-year risk score of CVD was calculated using the risk factors measured within six months of the lipid tests according to the Framingham Risk Assessment (FRS) chart for a 10-year risk of a total CVD event [4].

Variables required for CVD risk estimation, including comorbidities, level of blood lipids, level of systolic blood pressure, smoking status and medical history were extracted from the CPCSSN database. The most recent information regarding the lipid levels and the variables required for CVD risk estimation from every patient, was used in the event that the patient was visited multiple times during the study period.

### Study variables

LDL-C control is the primary outcome variable in this study. High-risk patients with a serum LDL-C level ≥2.0 mmol/L were considered as being poorly controlled, whereas those with lower LDL-C levels were regarded as being well controlled.

Independent variables, including age at the time of the lipid test, gender, place of residence, body mass index, smoking status, medical history of diabetes, hypertension and lipid lowering medication use were extracted from the database. Diabetes and hypertension were defined using CPCSSN algorithms for chronic conditions [17] which have high sensitivity, specificity and positive predictive value to detect these conditions with all scores being over 80–90 % [18, 19]. Obesity was defined as BMI ≥30; whereas those with BMI lower than 30, but higher than 25, were classified as overweight. The smoking status was extracted from the most recent record by the family physician at the time of the lipid test, and individuals were classified as non-smokers, past smokers, and current smokers. Place of residence (rural/urban) was determined using the first two characters of the postal code from each individual.

Lipid lowering medication (HMG-CoA reductase inhibitors, Fibrates, Bile Acid Sequestrants, Nicotinic Acid, and other agents) usage was classified into three categories as follows: 1) current user was defined as lipid-lowering medication use at the time of the lab test (started before the date of blood test and did not stop up to three months before the time of the blood test); 2) previous user was defined as the existence of any record of lipid-lowering medication use within two years from the date of the lab test and a record of discontinuation of at least three months before the date of the lab test; and 3) non-user was defined as no record of medication use within two years of the lab test. The type of lipid lowering medications was not specified in the analysis since more than 90 % of the medication users were on statins.

Patients with any record (diagnostic text, ICD code) of previous Ischemic Heart Disease (IHD), Peripheral Vascular Disease (PVD), or Cerebrovascular Disease (CVD) were categorized as those under secondary prevention. All other patients with no record of these conditions in the same period were regarded as those in need of primary prevention.

### Statistical analysis

Characteristics of the study population, as well as the mean and confidence intervals of the individual lipid components, were summarized using descriptive statistics. Classical tests of hypothesis, including the student’s *T*-test and the chi-squared test, were conducted to test for the association between variables. Logistic regression modeling was used to examine the association between LDL-C control status and possible influencing factors. Variable age was treated continuously. A *p*-value of less than 0.05 was considered statistically significant. All analyses were performed using STATA/SE 11.2 (Stata Corp., College Station, Texas, USA).

### Ethics

The study protocol was approved for ethics by the Health Research Ethics Authority (HREA) of Newfoundland and Labrador. Patients’ records and information were anonymous and de-identified prior to the analysis.
